# The History of Trachoma and Current Prevalence (Spotlight on Iran): A Review Article

**Published:** 2018-10

**Authors:** Gholamhossein YAGHOOBI, Gholamreza ANANI SARAB

**Affiliations:** 1. Dept. of Ophthalmology, Faculty of Medicine, Birjand University of Medical Sciences, Birjand, Iran; 2. Infectious Diseases Research Center, Birjand University of Medical Sciences, Birjand, Iran

**Keywords:** Trachoma, *Chlamydia trachomatis*, Prevalence, Iran

## Abstract

**Background:**

Trachoma as a common cause of infectious blindness is caused by *Chlamydia trachomatis*. This study aimed to review the available data from variety of sources and provide an overview of the epidemiological situation of Trachoma in Iran focused on the past seventy five years.

**Methods:**

A literature search of English and Farsi articles regarding trachoma in Iran from the electronic databases and paper documents was performed. Original articles, case reports and letters were included.

**Results:**

By the early and mid-20^th^ century, trachoma was widely endemic with the prevalence rate of more than 60% in Iran. Currently, trachoma prevalence is significantly lower than in the past and the elimination of trachoma is achievable in the near future. The decline in active disease is mainly attributed to improvement of socio-economic situation and personal and environmental hygiene rather than targeted interventions for epidemic control.

**Conclusion:**

Elimination of trachoma in Iran is achievable. However, trachoma prevalence estimation is required to be interpreted with some caution. Uncertainty around these estimates is partly because of the mismatch between the presence of infection and clinical findings.

## Introduction

Trachoma is causing infectious blindness and probably the third most common cause of blindness worldwide, after cataract and glaucoma (1). Indication of Trachoma has been frequently documented through the history. The oldest has found in China dating back to 2600 B.C. The first clear description of disease is from 1500 BC in the Ebers Papyrus which is a collection of medical prescriptions found in Egypt. There are also several written records from ancient Greece and Rome (2, 3).

Trachoma is a type of chronic keratoconjunctivitis caused by Gram-negative bacteria *Chlamydia trachomatis* (serotypes A–C) (4). Repeatedly infection, mainly found in childhood, progress to scarring complications and leading to visual loss and blindness in adulthood (5). The clinical manifestations of trachoma are subdivided into active (early) and cicatricial (late-stage) disease. Active disease, usually seen in childhood, is characterized by a chronic, recurrent follicular conjunctivitis. In later life, the chronic sequelae of trachoma included conjunctival scarring, trichiasis, and corneal scarring can lead to visual impairment (6,7). Although, Trachoma has been known as a global disease it has been eradicated in many parts of the world. Undeveloped parts of the world are still endemic for trachoma. Trachoma can be marked by poverty; inadequate personal hygiene, lack of safe water supplies and overcrowding are the facilitators of disease transmission (8). Active trachoma still persists in 50 countries around the world. The endemic areas are mainly in Africa, with the highest number, some poor rural areas in Latin America, the Western Pacific, Asia and Middle East (9, 10).

The disease even in the previous century was a global health concern but has now disappeared from developed countries. By gradual improvement in socioeconomic status, it is also largely declined in many developing countries as well as Iran (2). In addition to economicsituation, some widespread implementation of trachoma control programs played a major role in trachoma elimination and limiting it to some underdeveloped rural areas of developing world (11).

The objective of this paper was to review the data from variety of sources and display the analysis of the trachoma epidemiological situation in Iran from the past to the present.

## Methods

All article types including original articles, reviews and case reports related to trachoma disease and its history in Iran during the past seventy five years (1943 – 2017) were identified. In addition, data available either in Supplementary Material, abstracts or thesis was included. Sites claiming to index journals or articles published in Iran were searched. Some of the original articles, especially historical types were found in Farsi language, not ignored. All selected articles were read thoroughly by the authors to prepare a review on historical and current epidemiology of trachoma in Iran.

## Results

### The historical view of trachoma in Iran

Extensive searching of many data sources regarding the prevalence of trachoma revealed difficulties in obtaining consistent statistics on the number of trachoma sufferers. There is limited systematic collection of data in the field of trachoma prevalence in early and mid-20th century in Iran. The most available records about trachoma in Iran have been written in the second half of 20th and early 21st centuries and mainly focused on the pathophysiology, prevention and treatment strategies of the disease.

**Fig. 1:**
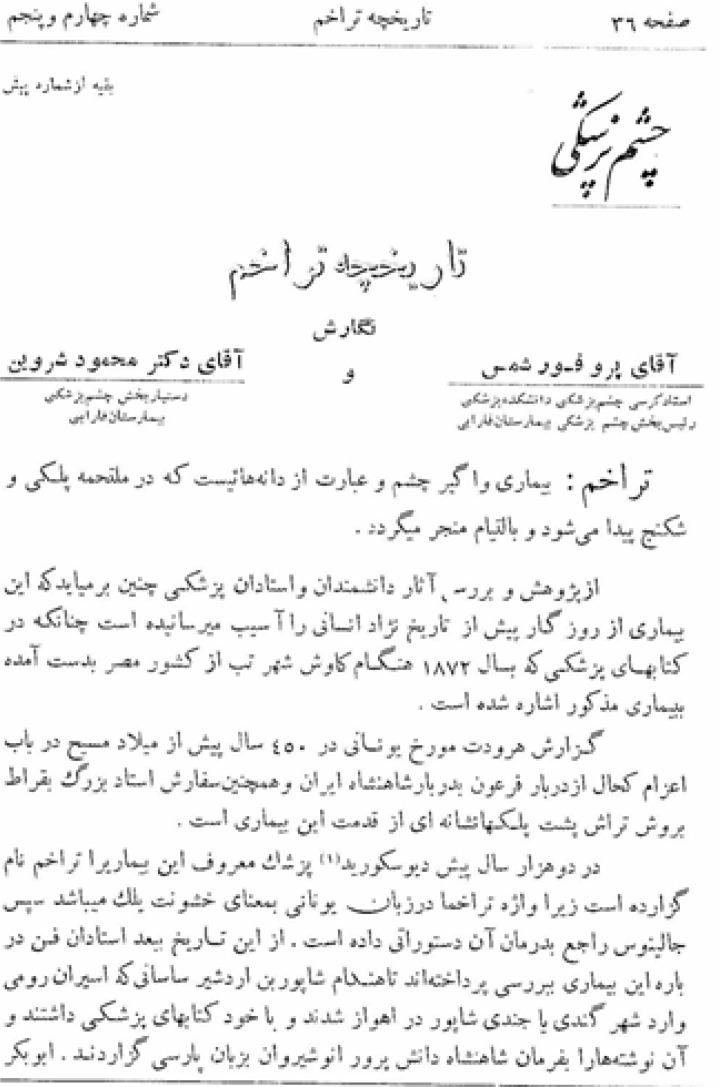
A page from an article published in Persian language about history of trachoma in 1943 (15)

Among the available information on the prevalence and distribution of trachoma in Iran, part of the story can be found in the oldest Iranian continually publishing university medical journal (12). Tehran University Medical Journals (TUMJ) started to publish scientific articles in Persian in 1943. The very first article about trachoma was brought out in the first volume, fourth issue of TUMJ by the well-known national ophthalmologist professor Mohammad Gholi Shams, involved in the elimination and treatment of trachoma in south and south-west of Iran from 1931(13,14). The article was mainly meant as a historical piece, talking about the general history of trachoma but relevant epidemiological data were not included (Fig. 1). In trachoma prevalence, he clings to his personal observation and estimated it as high as 60% in south-eastern provinces of Iran. Moreover, Shams stated about the influence of certain religious behavioral factors like hands and face wash before pray as the potential contributors in reducing trachoma incidence at that time (15). A series of articles on trachoma prevalence in Iran was started appearing in TUMJ in 1959. The first document was about the distribution and epidemiological status of trachoma and conjunctivitis in rural areas of Iran. The prevalence of trachoma in a total of 1270 inhabitants of rural areas in district of Malyer was 66.4% by 63.2% and 69% in males and females, respectively (16). One year later the second study from this group was published in TUMJ and is particularly notable for its clear statistics on trachoma in Iran. As far as is known this paper was a landmark in focus of public health, epidemiological thinking and using scientific method of documentation in Iran. The article included findings of a report contributed to the international conference of trachoma in Tunisia. The main findings of this study were as follows: active and cicatricial trachoma in northern areas (Caspian Sea coast), central area and in the south (Persian Gulf coast) was 41.7%, 49.8%, and 74% respectively and active trachoma was 13.4%, 19.3% and 38.6% for the mentioned areas in order (17). The report of epidemiological status of trachoma and other conjunctivitis in district of Dezful was published in TUMJ and the following information addresses the key results of the study. From 904 individuals were examined, 91% showed trachoma infection and 62% was diagnosed with active trachoma in average in rural areas. Trachoma prevalence, based on trachoma endemic data, was about 30%–40% in the capital of Tehran (especially in suburb of Tehran) (18).

Despite the irregular publication of this data, the above mentioned historical records provided a relatively informative picture from the epidemiological situation of trachoma in Iran at that time. The extremely high prevalence of trachoma was a serious health problem especially in rural areas in the late nineteenth and early twentieth century in Iran. The concern about trachoma led to legislation being passed that every medical student must attend and participate in 6 months eye disease programme for the completion of his/her medical training. Moreover, Professor Shams asked for help from Marks Ferline, the chairman of International Council of Ophthalmology, to send a group of ophthalmologists in order to treat and control trachoma in south of Iran. This was accepted and 3 Austrian ophthalmologists were sent to Iran before the Second World War began (14).

On the basis of published data from 1970sonwards, a marked decline in the incidence of trachoma has happened. A joint study was conducted by Institute of Ophthalmology in London and Tehran University in the late 1970s to provide quantitative data on the shedding of *C. trachomatis* in the eye secretion of patients. Cases included in this study, were from 3 high-prevalence villages in southern Iran, with various grades of active trachomatous or inactive hyperendemic trachoma. Conjunctival swabbings and eye secretions were collected in parallel from a total of 752 patients. In these patients, *C. trachomatis* was isolated in conjunctival swabbing in 57 (8%) and in eye secretion in 28 (4%) (19). A study was carried out over a 2-year period, on patients referred to eye medical center in Ahvaz in the south of Iran. In total of 1135 patient, blindness and low vision were diagnosed in 742 cases. Trachoma was the cause of visual problem in 10.9%. However, the study did not separate active trachoma from sequel of old inflammation (20). Trachoma was profoundly reduced compared to 1930 when Khuzestan was known as trachoma endemic district in Iran (21). A population-based cross-sectional study was determined the prevalence and causes of blindness and low vision in residents of Khuzestan Province. None of the cases was due to trachoma (22). Observations over a 5-yr period between 2004 and 2009 recorded 4.7% incidence of, trachoma keratopathy, among 1624 patients with a mean age of 41.3 ± 21.3 yr who underwent corneal transplantation in Labbafinejad Medical center in Tehran (23). In addition, the commonest chlamydial serotypes were mentioned in few studies. The serotyping tests were performed in the early 1970s by investigators at institute of ophthalmology in University of London. The tests were based on indirect immunofluorescence technique using anti-chlamydial sera obtained from patients in areas of hyperendemic trachoma in Iran. B and C were the most commonly chlamydial serotypes in these areas (24).

The statistics on the number of trachoma sufferers in Iran reveal the high prevalence of trachoma before oil and gas industry flourishing in 1970. A socioeconomic transformation resulted from the reinvestment of petrol income, took place rapidly in the last quarter of the 20th century in Iran. Increased national income and the Gross Domestic Product (GDP) per capita helped the development of urban infrastructures, safe water, community sanitation, transport, empowerment, and productivity as well as access to health and education. Overall hygiene and sanitation improvement in the country influenced the burden of many infectious diseases especially trachoma (8, 9). At present, it does appear to be an encouraging downward in the number of trachoma and its eventual elimination in Iran.

### Assessment of the burden of trachoma in Iran

Iran comprises 32 provincial regions which have a diverse range of climatic conditions from the tropical areas on the coast of the Persian Gulf to the deserts and arid lands on the Eastern half of the country. There are also wet and colder areas with woods in the North and West. The temperature range in some locations in Iran can be lower zero up to greater than 40 °C. In the past, when trachoma was of epidemic proportion, its distribution was not uniform in all regions of Iran. Warm areas like South and Central part of Iran were known as trachoma hyperendemic areas while residents in colder part of the country were not having trachoma as a serious health issue. Although a fresh calculation for the number of people blind or with low vision from trachoma has not been performed in very rare occasions there are old people, especially in hot climate areas, who have sequel of trachoma (25).

The trachoma control related SAFE strategy (Surgery/Antibiotics/Face-washing/Environmental change) has been recommended by WHO for the Global Elimination of Trachoma (GET) by 2020. SAFE elements describe an inclusive strategy of interventions and address the risk factors predisposing to disease transmission but must be integrated into existing national or regional health and development programs (26). Although, a national comprehensive trachoma control program has not been implemented in Iran successful health national strategies and the improvement in overall standards of living taken into account the impact of trachoma. We have done two studies on attendant patient of eye clinic in Birjand Medical University. Direct immunofluorescence assay (DFA) for Chlamydia infection was positive in 10% of patients with chronic conjunctivitis. The second study was done 10 yr later and no positive results were found, using PCR method for *C. trachomatis* among patients with chronic conjunctivitis. Although, the findings indicated hospital-based prevalence estimates of trachoma and cannot be correlated directly to the condition in general population the incidence ratio was much lower than the expected one (25, 27). A comparative study was carried out on 100 cases with vernal keratoconjunctivitis suspected to *C. trachomatis* infection and 100 controls that had refractive error without keratoconjunctivitis in Zahedan University of Medical Sciences. Chlamydial infection was detected by DFA in 4% (1 male and 3 female) and 1% (1 female) of cases and controls, respectively (28). In Mashhad Ghaem Hospital to go with clinical evidence of trachoma in different stages of the disease and Para-clinical data (three kinds of laboratory tests carried out for each). Within one year, 100 patients with clinical signs of trachoma and corresponding laboratory test results were recorded. Of them, 24%, 47%, and 85% were positive for inclusion body, DFA and cell culture respectively (29). A study carried out to do rapid assessment of trachoma in four southern provinces of Iran in 2004. In total, 5328 cases including 4782 children aged 1–9 yr and 546 adults were examined. Only in Sistan and Baluchistan Province, 7 cases of active follicular trachoma were observed (prevalence of 0.15%) with no cases of severe trachoma. Prevalence of trachoma in the studied areas was found to be less than the accepted level of WHO. Therefore, the results were promising and nationwide trachoma control program was not recommended as a health priority (30). However, caution is required around estimating the burden of trachomatous visual loss in Iran. A comprehensive study regarding the cause of blindness and low vision, paying particular attention to schools and community, would be required for the exact estimate of trachoma burden in Iran.

### Trachoma diagnostic and control challenges

In order to reduce the burden of trachoma, active disease must be diagnosed. The common diagnosis mainly is made on clinical ground and the laboratory tests can be used to assist in confirming the diagnosis. However, some caution is required in estimates of trachoma prevalence as uncertainty present on clinical signs, as a measure of trachoma prevalence. Clinical signs are sometimes poorly correlated with *C. trachomatis* infection, especially in low prevalence communities and those received mass treatment (2, 3).

### Clinical diagnosis

The clinical features are usually classified using the Simplified WHO Trachoma Grading System (Table 1) (31).

**Table 1:** WHO simplified trachoma grading classification system

***Abbreviation***	***Grade Description***
TF	Follicular trachoma: The presence of five or more follicles (.0.5 mm) in the upper tarsal conjunctiva
TI	Inflammatory trachoma: Pronounced inflammatory thickening of the tarsal conjunctiva that obscures more than half of the deep normal vessels
TS	Trachomatous scarring: The presence of easily visible scarring in the tarsal conjunctiva
TT	Trachomatousstrichiasis: At least one lash rubs on the eyeball
CO	Corneal opacity: Easily visible corneal opacity over the pupil

### Laboratory tests

There are techniques used to diagnose infection with *C. trachomatis* in the laboratory. These include cytological examination of stained slides of conjunctival swabs, growing the organism in tissue cultured cells or DNA-based nucleic acid amplification test by PCR (32). Recently, quantitative real-time PCR has been used to measure the relative load of infection in members of trachoma-endemic communities (33).

### Mismatch between clinical signs and laboratory tests

Survival and burden of the disease are significantly influenced by accurate diagnosis. There is a complex discrepancy between clinical picture and detection *Chlamydia*, active disease without chlamydial infections and conversely *chlamydia* detected in clinically normal individuals are quite common (1). There are some reasons for this mismatch. For example, while infection is present but signs of disease lags behind the resolution of infection during the incubation period by many weeks. The clinical diagnosis of active trachoma is not a reliable marker of infection in children and less reliable after treatment by antibiotic components. Therefore, pooling specimens by the DNA amplification tests to detect infection could be an appropriate strategy (34). However, when laboratory testing is not available, clinical examination of sentinel group especially monitoring of school children will be useful for monitoring and identifying endemic areas (6). The above mentioned complex mismatch between clinical signs and detection of *C. trachomatis* is a significant problem for trachoma control programmers (35). Moreover, the limited reliability of diagnostic tests should be considered. Controversy of diagnostic tools may result difficulty in estimating the actual Prevalence of diseases, particularly in low prevalence settings. Addressing such concerns may require effective trachoma intervention strategy to anticipate the prevalence of active infection and control the disease (35).

### The risk of re-appearing trachoma

The global water crisis is an environmental issue with the effects on control programs of infectious diseases such as trachoma (36). Since the 1990s, rainfall has been scarce in most parts of Iran, such that there is inadequate water required for crops, animals and human being in some areas across the country. In remote and dried rural areas people may restrict its use as much as possible and may neglect their personal hygiene. Insufficiency of personal hygiene due to scarcity of rain and water crises could create a basis for infectious diseases such as trachoma in these areas, where the infectious agent can arise and spread (37). Experiencing long-term water crisis implying that hygiene-health measures are required for monitoring the diseases caused by lack of personal hygiene(38).

## Conclusion

Because of the impoverished living conditions particularly in rural areas, trachoma was endemic in many parts of Iran in the early decades of the 20th century. Developments of many health essentials (hygiene, diagnosis, and treatment) along with socio-economic improvements resulted in sharp decline of trachoma in Iran. In spite of controversy exists in trachoma diagnosis, the elimination of trachoma is anticipated. Trachoma prevalence studies in Iran are promising because the available data pointed out the signs for elimination of this infectious disease. However, the black shadow of trachoma still is observable in the eyes of old people in some areas of Iran.

## Ethical considerations

Ethical issues (Including plagiarism, informed consent, misconduct, data fabrication and/or falsification, double publication and/or submission, redundancy, etc.) have been completely observed by the authors.
